# The Order of Concurrent Training Affects Acute Immunological Stress Responses and Measures of Muscular Fitness in Female Youth Judo Athletes

**DOI:** 10.1002/ejsc.12262

**Published:** 2025-01-31

**Authors:** Adrian Markov, Jens Bussweiler, Philipp Baumert, Norman Helm, Michael Rex, Sebastian Behm, Tom Krüger, Helmi Chaabene

**Affiliations:** ^1^ Faculty of Human Sciences Division of Training and Movement Sciences Research Focus Cognition Sciences University of Potsdam Potsdam Germany; ^2^ Olympic Testing and Training Center Brandenburg Potsdam Germany; ^3^ Research Unit for Orthopaedic Sports Medicine and Injury Prevention Institute for Sports Medicine, Alpine Medicine and Health Tourism Private University for Health Sciences Hall in Tirol Austria; ^4^ School of Sport and Exercise Sciences Liverpool John Moores University Liverpool UK; ^5^ Department of Sport Science Chair for Health and Physical Activity Otto‐von‐Guericke University Magdeburg Magdeburg Germany; ^6^ Institut Supérieur de Sport et de l’Education Physique du Kef Université de Jandouba Le Kef Tunisia

**Keywords:** adolescents, combat sports, leukocytosis, lymphocytosis, risk of infection, white blood cells

## Abstract

This study aimed to examine the acute effects of concurrent muscle strength and sport‐specific endurance exercise order on immunological stress responses, metabolic response, muscular‐fitness, and rating‐of‐perceived‐exertion (RPE) in highly trained youth female judo athletes. Thirteen female participants randomly performed two concurrent training (CT) sessions; strength‐endurance and endurance‐strength. Immune response, metabolic response, muscular fitness (i.e., countermovement jump‐derived force and power [CMJ‐force and CMJ‐power]), and RPE were measured at different time points (i.e., PRE, MID, POST, POST6h, and POST22h). There were significant time × order interactions for lymphocytes (*p* = 0.006, ES = 1.31), granulocyte–lymphocyte ratio (*p* = 0.002, ES = 1.56), and systemic inflammation index (*p* = 0.029, ES = 1.11), blood glucose and lactate (*p* < 0.001, ES = 2.09 and *p* = 0.0018, ES = 1.51, respectively), CMJ‐force (*p* = 0.033, ES = 1.26), and CMJ‐power (*p* = 0.007, ES = 1.40) as well as RPE (*p* < 0.001, ES = 2.05). CT‐induced acute (i.e., POST) but not delayed (i.e., POST6h and POST22h) order‐dependent immune cell count alterations in highly trained youth female judo athletes. All markers of the immune system went back to baseline values at POST22h. Metabolic responses were slightly higher following the endurance exercise (irrespective of the applied exercise order). CMJ‐measures and RPE fluctuated during both CT sessions but returned to baseline 6 h post‐exercise.

**Summary**

- Concurrent strength and sport‐specific endurance exercises induced acute (≤ 15 min) but not delayed (≥ 6 h) order‐dependent immune cell count alterations in highly trained youth female judo athletes. More particularly, the strength‐endurance order seems to have led to slightly higher acute immunological activation compared to the endurance‐strength order.
- All markers of the immune response taken post 22 h returned (or were close) to baseline values, suggesting a sufficient recovery from the exercise‐induced immune stress reaction within 22 h in female judo athletes.
- Metabolic responses (i.e., lactate, glucose) were slightly higher, following the endurance exercise (irrespective of the applied exercise order).

## 1 Introduction

The immune system plays a crucial role in protecting the body against various stressors, both internal and external, to maintain its overall function and health. Exercise is a common stressor that can disrupt cell homeostasis (Gleeson 2007; Walsh et al. 2011). One of the typical responses to exercise‐induced stress is leukocytosis, which refers to an increase in white blood cells (WBC) (Walsh et al. 2011). Leukocytosis is widely recognized as a marker of inflammation and infection (Opdenakker, Fibbe and Van Damme 1998). Earlier studies have demonstrated that both strength (Ihalainen et al. 2014; Nieman et al. 1995; Schlagheck et al. 2020) and endurance (Nielsen et al. 1996; Shek et al. 1995; Wahl et al. 2020) exercises can induce acute leukocytosis, and the magnitude of this response depends on factors such as exercise intensity, volume, and type (Bessa et al. 2016; Ghanbari‐Niaki, Saghebjoo and Hedayati 2011; Schlagheck et al. 2020; Walsh et al. 2011). Age and sex also play important roles, as individuals undergoing maturation, experience physiological changes in their tissues, organs, and body systems (DiFiori et al. 2014), which can make them prone to infection (Moreira et al. 2009; Nieman and Wentz 2019) and injury (Fabricant et al. 2016; Roberts 2014). However, there is a significant gap in the literature regarding the acute immunological responses to exercise in youth athletes, with only a limited number of studies conducted in this population (Freitas et al. 2016; Markov et al. 2023; Moraes et al. 2017; Puta et al. 2018).

Concurrent training (CT), which involves any combination of strength and endurance exercises, is a promising method to induce simultaneous adaptations in skeletal muscle and cardiovascular structures (Baar 2006; Coffey and Hawley 2017; Hickson 1980). However, the magnitude of these effects can vary depending on many factors (Fyfe, Bishop and Stepto 2014; Fyfe and Loenneke 2018; Ihalainen et al. 2017). One such factor is the order in which exercises are performed (i.e., strength‐endurance vs. endurance‐strength) (Coffey and Hawley 2017; Schumann et al. 2013; Taipale et al. 2014). Generally, the underlying immunological events of CT are rarely discussed. The few available studies indicate that the immune system‐related responses to CT depend on the design of the CT session (Enright et al. 2018; Inoue et al. 2016; Markov et al. 2023; Schumann et al. 2013, 2014; Sparkes et al. 2020). As such, it is reasonable to assume that the order of exercises may impact the immune system response. In a recently published study (Markov et al. 2023), we investigated the acute (< 15 min) and delayed (> 6 h) effects of exercise order on white blood cell (WBC) kinetics in male youth high‐performance judo athletes. Our findings revealed that when power and sport‐specific endurance exercises were combined within a single training session, performing power exercise before endurance exercise resulted in higher increases in WBC, lymphocytes (LYM), and granulocytes (GRAN) immediately after exercise and 6 h post‐exercise, compared to performing endurance exercise before power exercises.

Although CT is commonly used in team and individual sports to develop both cardiorespiratory endurance and muscle strength, there is a lack of research specifically focusing on female athletes' physical and physiological responses to CT. The existing studies in this area primarily focus on male athletes, leaving female athletes underrepresented (Costello, Bieuzen and Bleakley 2014; Lew et al. 2022; Patel et al. 2021). Furthermore, to the best of our knowledge, there are no previous studies in the literature regarding the effects of CT exercise order on the markers of the immune system stress response in youth female athletes. This points to a notable gap in the literature considering that youth female athletes, such as their male counterparts, are often exposed to high training loads, which can increase the risk of injury and infection (DiFiori et al. 2014; Fabricant et al. 2016; Roberts 2014). It is also important to note that physiological responses to exercise cannot be generalized from males to females because of their distinct biological characteristics (Landen et al. 2021). Accordingly, there is a need for further investigation into the specific immune responses of female athletes to CT exercise order to better understand and optimize their training outcomes.

Therefore, the primary objective of this study was to examine the effect of CT exercise order, specifically strength‐endurance versus endurance‐strength, on acute (< 15 min) and delayed (> 6 h; ≤ 22 h) immunological stress responses in youth female judo athletes. Additionally, we aimed to explore the effects of CT exercise order on measures of metabolic response, physical performance (i.e., muscle power), and rating of perceived exertion (RPE). Building on the findings of our previous study (Markov et al. 2023), we hypothesized that the exercise order would influence both the acute and delayed immune responses. Furthermore, we anticipate that changes in RPE and measures of muscular fitness would be dependent on the specific exercise order employed in the CT protocol.

## 2 Methods

### 2.1 Participants

Based on the study by Markov et al. (Markov et al. 2023), an a priori power analysis was conducted with a Type I error rate of 0.05% and 80% statistical power. The analysis indicated that 7 participants would be sufficient to detect a significant time × order interaction effect (Cohen's *d* = 0.95 for WBC). To account for potential attrition and to achieve better statistical power, the entire group of youth female judo athletes affiliated with a national training center was recruited for this study (*n* = 15). The general characteristics of the participants at baseline are presented in Table 1. All participants were engaged in regular training, attending a minimum of two sessions per day for at least 5 days a week. Additionally, they were actively involved in elite‐level competitions, indicating a high level of training and performance (McKay et al. 2022). Individuals with acute injuries or those reporting infectious diseases before and during the entire experimental period were excluded from the study. The maturity status of the participants was assessed using the maturity offset method for females developed by Mirwald et al. (2002). Because of sickness during the wash‐out period and missing data, two individuals were excluded from the experiment, leaving a final sample size of 13 females who completed the entire protocol. Prior to participation, written informed consent was obtained from both the legal guardians and the participants. The experimental procedure was approved by the Human Ethics Committee at Potsdam University (No. 45/2020) and the study was conducted in accordance with the relevant guidelines and regulations per the latest Declaration of Helsinki.

**TABLE 1:** The characteristics of participants.

Characteristics	Value
Number of participants	13
Age (years)	14.4 ± 2.1
Maturity offset (years)	2.0 ± 1.2
Sitting height (cm)	85.9 ± 3.6
Standing height (cm)	162.9 ± 6.3
Body mass (kg)	57.1 ± 10.7
Training age (years)	7.3 ± 1.3
Training volume (hours/week)	17 ± 4
1‐RM per kilogram body mass (kg)	2 ± 0.5
1‐RM range^a^ (kg)	85 to 245

Abbreviations: 1‐RM, one‐repetition maximum; cm, centimeter; kg, kilogram.

^a^ By reason of different weight categories.

### 2.2 Procedure

**FIGURE 1:**
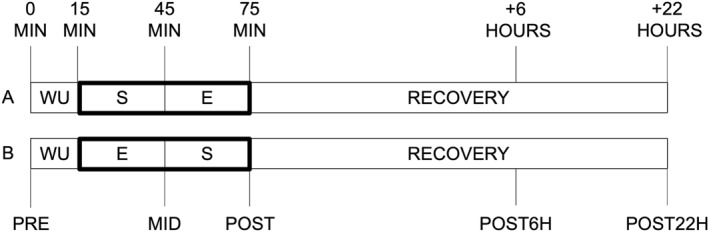
Schematic overview of the protocol. A, strength‐endurance order; B, endurance‐strength order; E, sport‐specific endurance exercise; MIN, minutes; S, strength exercise; WU, warm‐up.

All participants attended a total of four experimental sessions in the training/testing area. During the first two sessions, participants were introduced to the study design and familarizised with all exercises included in the protocol (refer to Section 2.3 for more details). Additionally, participants' one‐repetition maximum test for the leg‐press machine was conducted to assess their strength levels. In session three, participants were randomly assigned to either the strength‐endurance or endurance‐strength exercise order. Following a wash‐out period of 2 weeks, during which all participants maintained their regular training routine, the protocol was repeated. In session four, participants who completed the strength‐endurance order in session three now performed the endurance‐strength order, and vice versa. We instructed all participants to refrain from any form of exercise for 36 h before the two testing sessions. On the day of the experiment, participants were advised to have breakfast as part of their normal feeding routine. Due to COVID‐19 restrictions, participants were assigned to three groups. The first arrived at 7:00 a.m., the second at 9:30 a.m., and the third at 11:30 a.m. Before the experiment, all participants underwent a standardized warmed‐up protocol based on selected exercises from the FIFA 11+ program (Bizzini and Dvorak 2015). Following the experiment, participants resumed their daily routine but were restricted from engaging in any further physical activity until the final blood measures were taken on the next day (i.e., ≤ 22 h). For a visual representation of the experimental procedure, please refer to Figure 1.

### 2.3 Muscle Strength and Sport‐Specific Endurance Exercises

For the strength exercise, participants used a leg‐press machine (SCHNELL, Peutenhausen, Germany) and performed 4 sets at 70%–80% of each participant's one‐repetition maximum. Participants aimed to complete as many repetitions until volitional failure within each set. To maximize muscle time under tension (Burd et al. 2012), each set lasted a minimum of 45 s. Rest periods between sets were approximately 4 min. As for the sport‐specific endurance exercise, we employed the well‐established Special‐Judo‐Fitness‐Test (Sterkowicz, Zuchowicz and Kubica 1999). Each participant completed a total of four rounds of the test, with each round consisting of three sets (*A* = 15 s; B and C = 30 s) and a 10 s rest between sets. There was a break of 4 min between each round. During the test, participants ran between two partners positioned 12 m apart and aimed to execute the *ippon‐seoi‐nage* technique (Franchini et al. 2011) to throw each partner as many times as possible. In this context, partners were allocated based on the individual's weight category. The total duration, including breaks and post‐measures, for each of the strength and endurance exercises was around 25 min. This setup was predetermined in consultation with the coaching staff and had been previously used by the athletes.

### 2.4 Data Collection

Data (i.e., immune/metabolic markers, RPE, and jump performance) were collected at five different time points (Figure 1): PRE (i.e. right before the warm‐up), MID (i.e., at 45 min between the two exercises), POST (i.e., immediately after the end of the concurrent training sequence), POST6h, and the following morning (i.e., POST22 h). Capillary blood for immune response markers were obtained from the earlobe (20 μL). It should be noted that we did not collect immune response data between the strength and sport‐specific endurance protocol (i.e., MID) to avoid lengthening the break between the two exercises. All immune response markers, including WBC, LYM, GRAN, middle‐sized‐cells [MONO], and platelets, were analyzed immediately after blood sampling using a hematology analyzer system (Medonic M32, Boule Medical AB, Sweden). Intra‐assay coefficients of variability for micro pipette adapters were recently provided by the manufacturer (WBC ≤ 2.5%, PLT ≤ 3.0%, Boule Medical AB, Sweden). The system was used by several studies (Markov et al. 2023; Puta et al. 2018; Steidten et al. 2021) and operates based on the principle of impedance using a WBC discriminator. GRAN includes neutrophils, basophils, and eosinophils, whereas MONO is an estimation based on the total number of GRAN and LYM. Additionally, we calculated the granulocyte–lymphocyte ratio (GLR) and the systemic inflammation index (SII) based on the literature (Buonacera et al. 2022; Steidten et al. 2021):

$$\text{GLR}=\frac{\text{GRAN}}{\text{LYM}}$$

$$\text{SII}=\text{Platelets}\times \frac{\text{GRAN}}{\text{LYM}}$$

To measure blood lactate and blood glucose levels, an additional 10 μL of capillary blood was obtained from the earlobe at PRE, MID, and POST and analyzed by using a Biosen S‐Line device (EKF‐Diagnostics, Germany). RPE was assessed using the 6‐20‐BORG scale (Williams 2017). Based on the recommendations of Petrigna and colleagues (Petrigna et al. 2019), CMJ height, force, and power were evaluated using a force plate (Leonardo Jumping Platform, Novotec, Germany). During the CMJ, participants started from a standing position and performed a fast downward movement by flexing the knees and hips before rapidly extending the legs and performing a maximal vertical jump. During the test, participants were instructed to maintain their arms akimbo. The best result of the two consecutive repetitions was used for data analysis. Both RPE and jump performance were measured at PRE, MID, POST, and POST6h.

### 2.5 Statistical Analyses

To examine the effects of CT exercise order on the dependent variables, a repeated measure analysis of variance (ANOVA) was computed using a 2 (strength‐endurance vs. endurance‐strength) * 3 (time: PRE, MID, and POST) or 4 (time: PRE, MID, POST, and POST6H or PRE, POST, POST6H, and POST22H) factorial design (St and Wold 1989). Prior to the analysis, the normal distribution of the data was confirmed using the Shapiro–Wilk test (Ghasemi and Zahediasl 2012). In cases where sphericity assumptions were violated, the degrees of freedom were adjusted using the Huynh–Feldt (*ε* > 0.75) or Greenhouse–Geisser (*ε* < 0.75) correction values for *ε* (Field 2009). In the presence of significant order × time interactions, Bonferroni pairwise comparisons were conducted (Cohen 1988; Field 2009). Delta changes were calculated between PRE‐to‐MID, PRE‐to‐POST, PRE‐to‐POST6H, and PRE‐to‐POST22H. Effect sizes were also calculated and interpreted as trivial (ES < 0.20), small (0.2 ≤ ES < 0.50), moderate (0.50 ≤ ES < 0.80), or large (ES ≥ 0.80) (Cohen 1988). The results are presented as mean ± standard deviation (SD). Statistical significance was set at *p* < 0.05. Data analysis was performed using the Statistical Package for Social Science (SPSS, Chicago, IL, USA, version 29.0).

## 3 Results

For more detailed information on estimated marginal mean values, standard deviation, effect sizes, and time × order interaction, please refer to Table 2. Baseline differences between the two exercise orders were examined for all values, and no significant differences were found. It is worth mentioning that all datasets, tables, and graphs are accessible online. Please refer to the section “Availability of data and material” for access to the available resources.

**TABLE 2:** Means (± standard deviations) for all outcome measures at PRE, MID, POST, POST6H, and POST22H across both concurrent training orders.

Variable	Condition	PRE	MID	POST	POST6H	POST22H	Condition	Time	Interaction, ES
WBC (10^3^/μL)	Strength‐endurance	8.27 ± 2.29		12.91 ± 4.76	12.84 ± 3.68	6.94 ± 2.07	F_1, 12_ = 1.77; *p* = 0.208	F_2.74, 32.87_ = 21.82; ***p* *<* 0.001**	F_2.71, 32.55_ = 2.24; *p* = 0.107, ES = 0.87
	Endurance‐strength	9.03 ± 1.93		11.90 ± 3.52	14.42 ± 3.62	7.83 ± 2.05			
GRAN (10^3^/μL)	Strength‐endurance	4.30 ± 1.59		7.31 ± 2.90	8.71 ± 2.82	3.68 ± 1.32	F_1, 12_ = 1.64; *p* = 0.225	F_2.69, 32.33_ = 36.29; ***p* *<* 0.001**	F_2.35, 27.04_ = 3.26; *p =* 0.750, ES = 0.33
	Endurance‐strength	4.56 ± 0.66		8.19 ± 2.83	9.31 ± 2.50	3.84 ± 1.07			
LYM (10^3^/μL)	Strength‐endurance	3.42 ± 0.92		4.76 ± 2.91^a^	3.25 ± 0.78	2.78 ± 0.86	F_1, 12_ = 0.31; *p* = 0.585	F_2.20, 26.34_ = 1.00; *p =* 0.389	F_2.77, 33.27_ = 5.15; ***p =* 0.006**, ES = 1.31
	Endurance‐strength	3.82 ± 1.44		3.02 ± 1.27^a^	4.10 ± 1.41	3.54 ± 1.36			
MONO (10^3^/μL)	Strength‐endurance	0.55 ± 0.16		0.84 ± 0.36	0.87 ± 0.28	0.48 ± 0.12	F_1, 12_ = 2.06; *p* = 0.177	F_2.88, 34.58_ = 14.05; ***p* *<* 0.001**	F_2.66, 31.99_ = 3.72; ***p* = 0.035**, ES = 1.06
	Endurance‐strength	0.65 ± 0.21		0.69 ± 0.24	1.02 ± 0.30	0.57 ± 0.16			
GLR (10^3^/μL)	Strength‐endurance	1.31 ± 0.48		1.84 ± 1.09^a^	2.83 ± 1.12	1.35 ± 0.35	F_1, 12_ = 0.52; *p* = 0.486	F_1.86, 22.27_ = 19.01; ***p* *<* 0.001**	F_2.43, 29.20_ = 7.32; ***p* = 0.002**, ES = 1.56
	Endurance‐strength	1.33 ± 0.49		3.02 ± 1.23^a^	2.44 ± 0.92	1.20 ± 0.55			
SII (10^3^/μL)	Strength‐endurance	216 ± 103		301 ± 203^a^	461 ± 166	183 ± 66	F_1, 12_ = 0.02; *p* = 0.887	F_1.91, 22.88_ = 16.11; ***p* *<* 0.001**	F_2.51, 30.06_ = 3.66; ***p* = 0.029**, ES = 1.11
	Endurance‐strength	209 ± 112		433 ± 209^a^	389 ± 143	173 ± 76			
LA (mmol/L)	Strength‐endurance	0.96 ± 0.22	4.33 ± 1.64^a^	6.00 ± 4.15			F_1, 12_ = 0.74; *p =* 0.408	F_1.15, 13.85_ = 36.19; ***p* *<* 0.001**	F_1.14, 13.62_ = 6.83; ***p =* 0.018**, ES = 1.51
	Endurance‐strength	0.88 ± 0.26	6.35 ± 2.95^a^	4.62 ± 1.81					
GLU (mg/dL)	Strength‐endurance	4.73 ± 0.50	4.46 ± 0.42^a^	5.60 ± 1.27^a^			F_1, 12_ = 0.64; *p* = 0.438	F_2, 24_ = 1.40; *p* = 0.266	F_1.76, 21.06_ = 13.08; ***p* *<* 0.001**, ES = 2.09
	Endurance‐strength	4.81 ± 0.54	5.69 ± 1.27^a^	4.66 ± 0.65^a^					
CMJ‐H (cm)	Strength‐endurance	32.26 ± 3.20	30.81 ± 3.88	29.97 ± 4.65	31.86 ± 3.06		F_1, 12_ = 9.67; ***p* = 0.009**	F_3, 36_ = 3.14; ***p* = 0.037**	F_1.84, 22.02_ = 1.17; *p* = 0.326, ES = 0.63
	Endurance‐strength	32.50 ± 2.68	33.23 ± 4.07	32.11 ± 3.15	33.36 ± 4.15				
CMJ‐P (W)	Strength‐endurance	2.17 ± 0.57	2.13 ± 0.55^a^	2.15 ± 0.42	2.17 ± 0.45		F_1, 12_ = 2.40; *p* = 0.147	F_2.12, 25.41_ = 2.81; *p* = 0.076	F_2.11, 25.33_ = 5.91; ***p* = 0.007**, ES = 1.40
	Endurance‐strength	2.19 ± 0.93	2.27 ± 0.50^a^	2.06 ± 0.47	2.24 ± 0.49				
CMJ‐F (N)	Strength‐endurance	1.34 ± 0.35	1.28 ± 0.32^a^	1.35 ± 0.33	1.31 ± 0.32		F_1, 12_ = 1.51; *p* = 0.243	F_1.34, 16.49_ = 1.07; *p* = 0.340	F_1.41, 16.90_ = 4.75; ***p* = 0.033**, ES = 1.26
	Endurance‐strength	1.32 ± 0.34	1.35 ± 0.31^a^	1.17 ± 0.43	1.31 ± 0.31				
RPE	Strength‐endurance	7 ± 1	14 ± 2^a^	17 ± 2^a^	9 ± 3		F_1, 12_ = 0.38; *p* = 0.551	F_2.95, 35.45_ = 152.41; ***p* *<* 0.001**	F_3, 36_ = 12.62; ***p* *<* 0.001**, ES = 2.05
	Endurance‐strength	8 ± 2	17 ± 2^a^	15 ± 2^a^	8 ± 2				

*Note:* Values in bold highlight significant effects.

Abbreviations: CMJ‐F, countermovement jump force; CMJ‐H, countermovement jump height; CMJ‐P, countermovement jump power; ES, Effect size; GLR, granulocyte–lymphocyte ratio; GLU, glucose; GRAN, granulocytes; LA, blood‐lactate; LYM, lymphocytes; MID, in between the protocol; POST, immediately after the protocol; POST22H, twenty‐two hours after the protocol; POST6H, six hours after; PRE, before the protocol; RPE, rate of perceived exertion; SII, systemic inflammation index; WBC, white blood cells.

^a^ Significant time × order interaction effect.

### 3.1 Blood Markers of Immune Response

**FIGURE 2:**
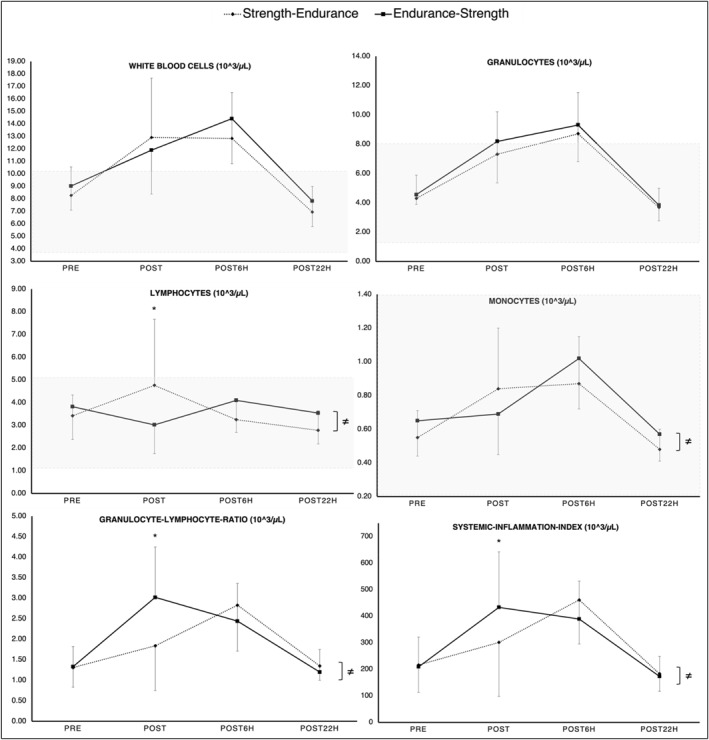
Means and standard deviation for all immunological blood markers measured at PRE, POST, POST6H, and POST22H for the strength‐endurance (dashed line) and endurance‐strength order (solid line). The graph highlights that concurrent training induced order‐dependent immune cell count alterations in healthy youth female judo athletes. From an acute (≤ 15 min) perspective, significant differences between the two exercise orders in lymphocytes, monocytes, granulocyte–lymphocyte ratio, and the systemic inflammation index were observed. From a delayed (≤ 6 h) perspective, there were no significant differences. Shaded zone marks lower and upper reference values provided by the manufacturer (Medonic M32 series). ≠, stands for overall significant time × order interaction effect. *, stands for significant difference at the respective time point.

Findings indicated significant time × order interactions for LYM, MONO, GLR, and SII. For LYM, the post‐hoc analysis indicated significantly larger PRE‐to‐POST increases in strength‐endurance compared to endurance‐strength. Regarding GLR and SII, findings indicated significantly larger PRE‐to‐POST increases for endurance‐strength, compared to strength‐endurance. No significant differences were observed between PRE‐to‐POST6H, and PRE‐to‐POST22H for all parameters, irrespective of the exercise order. A graphical representation of these results can be found in Figure 2.

### 3.2 Metabolic Response

**FIGURE 3:**
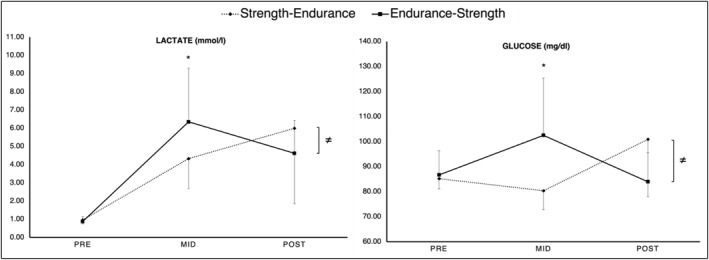
Means and standard deviation for all metabolic values collected at PRE, MID, and POST for the strength‐endurance (dashed line) and endurance‐strength order (solid line). The graph highlights significant time × order interaction effects with significantly larger PRE‐to‐MID increases in blood glucose and lactate following the endurance exercise (as part of the endurance‐power order) compared to significantly larger PRE‐to‐POST increases in blood glucose following the endurance exercise (as part of the power‐endurance order). ≠, stands for overall significant time × order interaction effect. *, stands for significant difference at the respective time point.

Our findings indicated significant time × order interactions for blood glucose and lactate. Results showed significantly larger PRE‐to‐MID increases in blood glucose and lactate, following the endurance exercise compared to the strength exercise. From PRE‐to‐POST, changes in blood glucose were significantly larger for the strength‐endurance order, compared to the endurance‐strength order. A graphical representation of these results can be found in Figure 3.

### 3.3 Physical Performance and Rating‐of‐Perceived‐Exertion

**FIGURE 4:**
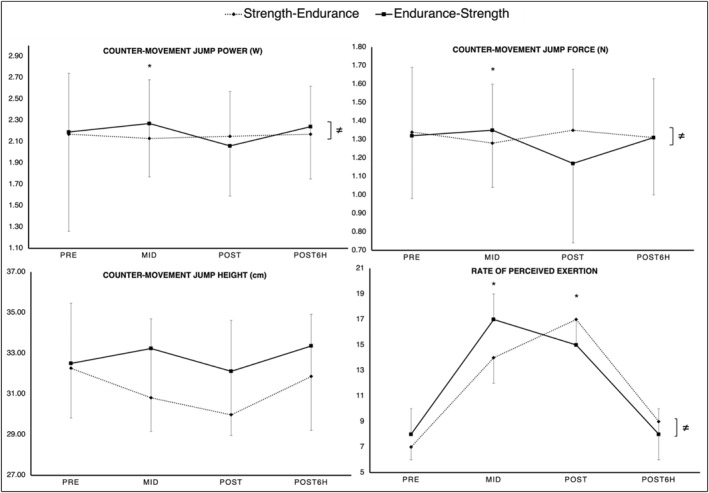
Means and standard deviation for all physical performance and perceived exertion values collected at PRE, MID, POST, and POST6H for the strength‐endurance (dashed line) and endurance‐strength order (solid line). The graph highlights significant time × order interactions for CMJ‐performance and rate of perceived exertion. CMJ‐force and CMJ power showed significantly larger PRE‐to‐MID performance increases, following the endurance‐strength order. Rate of perceived exertion was significantly higher following the endurance exercise, irrespectively of the applied exercise order. ≠, stands for overall significant time × order interaction effect. *, stands for significant difference at the respective time point.

For the physical performance, a significant time × order interaction was observed for CMJ‐power and force with significantly larger PRE‐to‐MID performance increases for the endurance‐strength order, compared to the strength‐endurance order. Regarding RPE, there was a significant time × order interaction with larger PRE‐to‐MID values, following the endurance exercise compared to the strength exercise. Additionally, RPE values were significantly larger from PRE‐to‐POST, following the strength‐endurance order, compared to endurance‐strength. A graphical representation of these results can be found in Figure 4.

## 4 Discussion

The main purpose of this study was to investigate the effect of CT exercise order, specifically strength‐endurance versus endurance‐strength, on acute (< 15 min) and delayed (> 6 h; ≤ 22 h) immunological stress responses in youth female judo athletes. As a second aim, we sought to explore the effects of CT exercise order on measures of metabolic response, physical performance (i.e., muscle power), and RPE. Results indicated that CT generated acute (≤ 15 min) but not delayed (≥ 6 h; ≤ 22 h) order‐dependent alterations in immune cell count in female youth judo athletes. More particularly, findings indicated an order effect for LYM, MONO, GLR, and SII, whereas WBC and GRAN did not show any order‐dependent effects at all the time points. Regarding metabolic response and physical performance, the results indicated acute order‐dependent alterations (< 15 min) that returned to baseline values 6 h after exercise.

### 4.1 Blood Markers of the Immune Response

It is widely accepted that physical exercise enhances immune system activity (Valencia‐Sánchez et al. 2019), resulting in changes in the number of peripheral immune cells in a time‐specific manner (e.g., leukocytosis and lymphocytopenia). Therefore, our results show representable cell kinetics within both exercise orders. However, based on our recently published paper (Markov et al. 2023), we expected an order effect, particularly in WBC and GRAN. Accordingly, we previously reported an acute (≤ 15 min) and delayed (≤ 6 h) order effect for WBC and GRAN (Markov et al. 2023). Meanwhile, the current study indicated that concurrent strength and sport‐specific endurance exercises induced similar WBC, and GRAN alterations from PRE‐to‐POST and PRE‐to‐POST6H in youth female judo athletes, irrespective of the applied exercise order. In the strength‐endurance order, WBC and GRAN increased from PRE‐to‐POST (∆56%, 70%, respectively), and from PRE‐to‐POST6h (∆55%, 103%, respectively). Similarly, in the endurance‐strength order, WBC, and GRAN increased from PRE‐to‐POST (∆32%, 80%, respectively), and from PRE‐to‐POST6h (∆60%, 104%, respectively). In fact, our previous study markedly differed from the current one. Here, we included less participants, female athletes only, while considering one additional parameter of immune response (i.e., MONO), as well as one additional time point of measurement (i.e., POST22H). Moreover and probably most importantly, the applied strength exercise in the current study aimed to induce muscle hypertrophy effects (i.e., 4 sets of maximum number of repetitions at 70%–80% of one‐repetition maximum). Meanwhile, in our previous study (Markov et al. 2023), we aimed for the development of muscular power (i.e., 4 sets of 8 repetitions at 30%–40% of one‐repetition maximum). As such, a comparison between these two investigations is challenging although the study design was very similar.

However, we would suggest that the distinct results between these two studies are most likely because of the different strength exercises applied (i.e., power vs. strength). Generally, we would argue that minimal changes within the design of a CT session significantly affects the magnitude of CT‐induced muscle damage, which in turn causes distinct immune responses. In this context, previous literature described muscle damage as a result of mechanical stress and impaired calcium homeostasis (Chatzinikolaou et al. 2010) with a direct impact on performance capacity, muscle soreness, and force production (Owens et al. 2019), which is closely tied to inflammatory responses and leukocyte accumulation (Chazaud 2020; Paulsen et al. 2012). Nonetheless, this assumption remains speculative since we did not collect any measures of muscle damage. This should be a topic of future research.

Concerning LYM, there was a significant time × order interaction from PRE‐to‐POST. Accordingly, LYM increased from PRE‐to‐POST by roughly one‐third (∆39%) in the strength‐endurance order compared to a slight decrease (∆21%) within the endurance‐strength order. The increase in LYM after the strength‐endurance order is most likely due to an exercise‐induced mobilization of lymphoid immune cells into the bloodstream (Campbell and Turner 2018; Rooney et al. 2018). Meanwhile, the decrease in LYM right after the endurance‐strength order indicates an increased demand for immune cells within peripheral tissue (Campbell and Turner 2018; Krüger et al. 2008; Kruger and Mooren 2007). In this regard, we did not measure immune cell kinetics between the strength and endurance exercises (i.e., MID). Thus, considering the transient nature of LYM fluctuations, it is highly probable that we have missed the time point at which LYM levels were elevated following the endurance‐strength order. Albeit not statistically significant, we observed a slight increase in LYM from POST‐to‐POST6H, following the endurance‐strength order compared to the strength‐endurance order. Additionally, it is important to note that LYM values observed POST22H were slightly below those registered at PRE following both exercise orders. This can potentially be explained by the exercise‐induced migration of lymphocytes from the bloodstream to peripheral tissues to enhance immune surveillance, as suggested in previous research (Campbell and Turner 2018; Krüger et al. 2008; Kruger and Mooren 2007).

MONO account for the innate immune system, are made up of myeloid stem cells, and mature within the bone marrow. In response to increased and sustained muscle contraction, MONO immigrate to skeletal muscles and differentiate into M1‐ and M2‐macrophages (Chazaud 2020; Julier et al. 2017; Tidball 2017). This process directly links the inflammatory process with muscle regeneration (Tidball 2017) and highlights the importance of MONO in the context of exercise. Regarding our results, there was a significant time × order interaction for MONO. Accordingly, the strength‐endurance order induced increases from PRE‐to‐POST (∆53%) and PRE‐to‐POST6H (∆58%). Meanwhile, the endurance‐strength order induced only marginal changes from PRE‐to‐POST (∆6%) but markedly increases from PRE‐to‐POST6H (∆57%). From PRE‐to‐POST22H, MONO felt below baseline, irrespective of the applied exercise order (∆13%). Our results indicate that the strength‐endurance order causes a higher immediate immune response/activation compared to the endurance‐strength order. Although, at POST6H, comparable high levels of inflammatory response were observed for both exercise orders. In addition, from PRE‐to‐POST22H, we found MONO levels slightly below baseline values, following both exercise orders. It should be mentioned though that MONO were measured indirectly based on the number of middle‐sized‐cells. Despite this, our results seem of great importance given the critical role of MONO in muscle repair and growth (Tidball and Villalta 2010).

GLR and SII increased from PRE‐to‐POST more than one‐fold (∆127% and 107%, respectively) within the endurance‐strength order, whereas the strength‐endurance order induced significantly lower values (∆40% and 39%, respectively). From a delayed perspective (i.e., PRE‐to‐POST6h), the strength‐endurance order induced greater but not statistically significant increases in GLR compared to the endurance‐strength order (∆116% and 83%, respectively). In clinical research, GLR and SII are commonly used as a marker of disease (Buonacera et al. 2022). Due to their high correlations with other inflammatory markers (Huang et al. 2018; Islas‐Vazquez et al. 2020; Zhu et al. 2020) such as the C‐reactive‐protein and Interleukin‐6, a consideration of GLR and SII seems to be important. With respect to CT, the available literature is sparse. Although it was previously shown that CT in general, induces GLR increases lasting for up to 3 h (Bessa et al. 2016). In the current study, we found significant time × order interaction effects for GLR and SII. Taken together, the results observed for LYM, GLR, and SII are in line with our previous findings (Markov et al. 2023).

### 4.2 Metabolic Response

With respect to the metabolic responses, our results showed significant time × order interactions for blood lactate and glucose. Accordingly, there were larger increases in blood glucose and lactate from PRE‐to‐MID following the endurance‐strength order, (∆ + 18% and ∆ + 621%, respectively) compared to the strength‐endurance order (∆ − 6% and ∆ + 351%, respectively). In addition, from PRE‐to‐POST, we observed that the strength‐endurance order induced larger increases in blood glucose and lactate (∆ + 18% and ∆ + 525%, respectively) compared to the endurance‐strength order (∆ − 3% and ∆ + 425%, respectively). Consequently, the endurance task seems to be the main driver of the observed metabolic responses. This is in line with the findings from our previous study (Markov et al. 2023).

### 4.3 Physical Performance and Rating‐of‐Perceived‐Exertion

Our findings indicated a significant order effect for CMJ‐force and CMJ‐power. Following the endurance‐strength order, muscular fitness values increased from PRE‐to‐MID, following the endurance exercise (∆2% and 4%, respectively) and decreased after the strength exercise (∆11% and 6%, respectively). Meanwhile, the strength‐endurance order induced performance decreases following the strength exercise (∆4% and 2%, respectively) but there are no marked differences compared to the baseline values after the endurance exercise (± ∆1%). Additionally, there was an order effect in RPE from PRE‐to‐MID and PRE‐to‐POST. Generally, regardless of the exercise order, the endurance exercise induced higher RPE values compared to the strength exercise. This would be in line with our recently published study (Markov et al. 2023). Overall, it seems that neither measures of muscular fitness nor RPE alone allow us to make definitive assertions about an athlete's physical condition. Therefore, considering the practical implications of the current study, practitioners should monitor internal and external measures of training load. Supported by the literature, this will help to properly guide recovery strategies (Balsalobre‐Fernández, Tejero‐González, and del Campo‐Vecino 2014; Cardinale and Stone 2006; García‐Pinillos et al. 2021; Halson 2014; Impellizzeri, Marcora and Coutts 2019; McLaren et al. 2018).

## 5 Limitations and Future Research Perspectives

This study has some limitations that should be discussed. First, because of the time‐consuming cell analyses, which took place immediately after blood collection, we did not obtain immune cell counts between the strength and sport‐specific endurance exercise (i.e., MID). Second, we did not measure menstrual cycle phases. In fact, a recent review from Notbohm et al. 2023 reported significantly higher resting values of GRAN and MONO but not LYM in the luteal phase compared to the follicular phase in healthy premenopausal women aged ≤ 45 years. Although we did not find any significant baseline differences across all immune markers it should be noted that some participants may have been tested within different menstrual cycle phases. Finally, it needs to be mentioned that we measured indirect markers of inflammation (i.e., GLR and SII). There is evidence that both GLR and SII present moderate‐to‐high correlations with other well‐established inflammatory markers such as C‐reactive protein and Interleukin‐6 (Huang et al. 2018; Islas‐Vazquez et al. 2020; Zhu et al. 2020) . Future research may investigate immune responses to repeated versus single exercise bouts throughout a defined microcycle (i.e., day and week) to see if constant WBC measures, for instance, could serve as a feasible tool to objectively control for training load. Despite that, we recommend future studies rely on more prominent markers (e.g., cytokines) to provide a more comprehensive and prominent inflammatory status. Taken together, to substantiate and expand the findings of the present study, future research should investigate different exercise settings, sexes, further time points of measurement, and additional objective markers of immune response (e.g., macrophages, and myokines) together with measures of muscular performance.

## 6 Conclusion

The main findings of this study indicated that concurrent strength and sport‐specific endurance exercises induced acute (≤ 15 min) but not delayed (≥ 6 h) order‐dependent immune cell count alterations in highly trained youth female judo athletes. More specifically, the strength‐endurance order seems to have led to slightly higher acute immunological activation compared to the endurance‐strength order. It is worth noting that at POST22H, all markers of the immune response returned (or were close) to baseline values. This particular observation suggests a complete recovery from the exercise‐induced immune stress reaction within 22 h in female judo athletes. Additionally, CMJ‐force and RPE fluctuated during both CT sessions but went back to baseline values 6 h post‐exercise. Generally, RPE indicated that the endurance exercise was more strenuous compared to the strength exercise. In this regard, metabolic responses (i.e., lactate and glucose) were slightly higher, following the endurance exercise (irrespective of the applied exercise order). These results are relevant for practitioners as they can assist in the optimal management of judo training load in youth female athletes.

## Conflicts of Interest

The authors declare no conflicts of interest.

## Data Availability

All data are freely available on repositories of the Open Science Framework (https://osf.io/snqkb/).
